# A Pediatric Patient with Severe Obstructive Sleep Apnea and Comorbid Depression and Substance Abuse

**DOI:** 10.1155/2023/9985503

**Published:** 2023-11-10

**Authors:** Caitlin E. Leconte, Joshua W. Ng, Ann M. Manzardo, Mitchell M. Douglass

**Affiliations:** ^1^University of Kansas School of Medicine, 2060 W 39th Avenue, Kansas City, KS 66103, USA; ^2^University of Kansas School of Medicine-Wichita, 1010 N Kansas Street, Wichita, KS 67214, USA; ^3^Department of Psychiatry and Behavioral Sciences, University of Kansas Medical Center, 3901 Rainbow Boulevard, Kansas City, KS 66103, USA

## Abstract

Obstructive sleep apnea (OSA), depression, and substance abuse problems share similar symptomatology and have significant interplay. An underlying diagnosis of OSA can often be overlooked in patients with significant psychiatric illness and polysubstance use. Pediatric OSA is often associated with adenotonsillar hypertrophy and frequently requires surgical intervention for resolution of symptoms. Untreated OSA can worsen mental status and encourage polysubstance abuse as a form of self-medication. Proper identification and management of OSA plays an important role in treating psychiatric conditions. We report a 16-year-old with major depressive disorder (MDD), suicide attempts, polysubstance use disorder, and severe OSA admitted to an inpatient psychiatric facility. History included sleep and mood disturbances started at age 12. Patient presented with apnea–hypopnea index greater than 50 and started on bilevel-positive airway pressure (BiPAP) prior to admission. Management of OSA led to significant improvement of MDD, insomnia, and polysubstance abuse. OSA can often be overlooked in patients with MDD or substance abuse. Among adolescent patients with poorly managed psychiatric conditions, significant sleep disturbances, and polysubstance abuse, providers should maintain a high degree of suspicion for OSA, as its proper management will aid in the management of the other conditions.

## 1. Introduction

Obstructive sleep apnea (OSA), depression, and substance use disorder (SUD) each present their own health challenges. Apnea from OSA has been related to neuroinflammatory injuries from cyclic hypoxemia [1], depression increases cardiovascular disease risk [2], and SUD is consistently associated with increased morbidity and mortality [3]. These three problems overlap. For example, 90% of patients with major depressive disorder (MDD) have some type of sleep problem and OSA often pairs with SUD in the form of self-medication [2, 4]. Additionally, rates of OSA, depression, and substance abuse have increased over the past few decades particularly in the pediatric population [5–8].

Pediatric OSA has several important differences when compared to adult OSA. Pediatric OSA is most commonly due to adenotonsillar hypertrophy because tonsillar tissue enlarges faster than surrounding airway structures. Thus, surgical intervention is often essential in pediatric cases [9]. Respiratory mechanisms to compensate for apnea differ between adults and pediatrics, too. Adults more readily exhibit autonomic stimulation to increase respiratory rate in response to apneic episodes; conversely, children have a higher arousal threshold, leading to a lower likelihood of appropriately responding when exposed to stressors such as hypoxemia during apneic sleep [10].

OSA predominantly occurs during rapid eye movement (REM) sleep. This stage of sleep is characterized by increased cholinergic neuron activity with decreased activity in adrenergic and serotonergic neurons; this results in total body paralysis of voluntary muscles apart from the extraocular muscles [11]. This paralysis includes the genioglossus muscle whose inhibition increases tendency for upper airway collapse. When apnea occurs, it is enough to fragment sleep and impair any REM sleep benefits [12]. REM sleep may play roles in facilitating cortical plasticity, restoring cell function, and even heightening creativity [13]; however, REM sleep is perhaps best-ascribed roles in memory consolidation, as evidenced by increased hippocampal and limbic activation during this sleep stage [14]. This is of note especially since children have a higher percentage of REM sleep compared to adults [9]. In fact, greater than half of all pediatric obstructive apneas occur during REM sleep even though REM occurs for less than a quarter of total sleep time [15].

In short, pediatric OSA has unique features from adult OSA which may make it more difficult to treat, and if untreated, it can leave lasting effects. Some complications may include long-term deficits in cognition such as lower IQ scores or impaired executive function [16].

## 2. Case Report

Ms. A is a 16-year-old female with a history of MDD, moderate, recurrent, SUD, and severe OSA admitted to our inpatient pediatric psychiatry facility for a second time within 1 year for suicidal ideations.

### 2.1. Psychiatric History

Psychiatric history is notable for post-traumatic stress disorder (PTSD), attention-deficit/hyperactivity disorder (ADHD), oppositional defiant disorder, obsessive–compulsive disorder, borderline personality disorder, and recurrent suicidal ideation. At age 12, the patient presented with symptoms of depressed mood, decreased interest, decreased appetite, impaired concentration, anhedonia, sleep disturbances, and suicidal thoughts consistent with a diagnosis of MDD. She was also noted to have symptoms consistent with ADHD with primary inattentive features. Additionally, she had significant anxiety symptoms with an emergency room visit confirming panic attacks as the etiology. All diagnoses were made utilizing diagnostic criteria from the Diagnostic and Statistical Manual of Mental Disorders, Fifth Edition (DSM-5). Over the next year, she attempted suicide twice by forearm laceration. At age 13, she attempted to overdose on bupropion; concerns were noted by her treating psychiatrist for borderline personality disorder in an adolescent. At age 15, she attempted overdose with tamsulosin. She began to see a psychiatrist specializing in borderline personality disorder following this overdose. In total, by age 16, she had been hospitalized eight times for suicidal behavior.

Psychosocial stressors included parental divorce and custody battle. She was diagnosed with PTSD relating to significant physical, sexual, and emotional abuse. She experienced unstable housing following her parents' divorce, living with her father, extended family, or at foster homes. At school, she experienced academic difficulties and bullying.

She had trialed many psychotropic medications with limited efficacy. Mood stabilizers including 50 mg quetiapine nightly, 10 mg aripiprazole daily, 100 mg lamotrigine daily, and 450 mg lithium nightly were trialed for management of irritability and dysregulated mood with no significant side effects or benefit. Antidepressants including 150 mg bupropion daily and 40 mg fluoxetine daily were trialed, only bupropion was found to improve mood. Thirty-six milligrams methylphenidate daily for ADHD was found beneficial for energy and attention.

### 2.2. Substance Use History

This patient's history was notable for polysubstance abuse since the age of 12. Illicit substances included stimulants, psychedelics, nicotine vapor products, and intranasal cocaine. At age 14, she was expelled from middle school due to psychedelic mushroom use. She had no legal charges associated with drug use. Marijuana was the patient's drug of choice. She began smoking early in the morning and smoked at least four bowls daily. The patient reported this substance use helped manage her suicidal ideations, fatigue, and sleep disturbances.

### 2.3. Medical History

In addition to severe OSA, medical history is notable for morbid obesity greater than the 130^th^ percentile for age group, 4+ tonsils with multiple episodes of tonsillitis and strep throat, and significant nasal scarring secondary to intranasal substance use. She was encouraged to make lifestyle changes; no medications were prescribed for her obesity. The patient had a history of significant sleep disturbances beginning at age 12. Her poor sleep resulted in significant mood problems and difficulties with interpersonal relationships. She had been expelled from school due to excessive daytime sleepiness. History notable for frequent nighttime headaches leading to arousal and distress. Trial of 2 mg prazosin did not improve sleep disturbances.

### 2.4. Presentation Prior to OSA Management

At age 15, the patient was admitted to our inpatient psychiatric facility with suicidal ideations and significant sleep disturbances. The patient described feelings of deep hopelessness and worthlessness. She reported problems with interpersonal relationships, impaired concentration, decreased interest in life, fatigue, anhedonia, anxiety, active suicidal thoughts, and diffuse body aches. She had stopped taking all psychotropic medications for several months, stating that they did not improve symptoms and only led to weight gain. Patient was using intranasal cocaine, marijuana, lysergic acid diethylamide, mushrooms, ketamine, and nicotine whenever she was able to obtain these substances. Urine drug screen was positive for cocaine and tetrahydrocannabinol (THC). She also reported heavy daily alcohol use. Snoring during admission was so significant that she was moved to a private unit. Staff noted apneic episodes and oxygen desaturations below 90% throughout the night. During admission, she was started on lithium carbonate 450 mg daily for mood stabilization. At time of discharge, she was referred to otolaryngology for evaluation and pulmonology for a sleep study.

### 2.5. Diagnosis with OSA

After discharge, the patient was diagnosed with severe OSA with apnea–hypopnea index greater than 50. Testing showed oxygen saturations below 88% with a nadir down to 63.5%. Adenotonsillectomy was discussed but declined. Specific concerns per the patient were postoperative pain, opiate exposure, vocal cord changes, and the likelihood patient would remain BiPAP dependent. She was fitted for a portable BiPAP machine with adequate symptom control at pressures of 20/10.

### 2.6. Current Admission

Patient's current admission occurred 10 months after diagnosis of OSA. She was admitted for recent suicidal statements in the setting of acute alcohol intoxication. She reported that her suicidal ideations had resolved once she was no longer intoxicated. She denied any active suicidal ideations or plans for self-harm at time of admission. Her depression was still present with symptoms of depressed mood, difficulty falling asleep, anhedonia, fatigue, feelings of worthlessness and hopelessness, poor concentration, and decreased appetite. However, she stated these symptoms had decreased in severity significantly compared to the previous admission. She denied any self-harm over the last several months. She reported significant improvement of mood and energy compared to the previous admission. She had discontinued all illicit drug use except marijuana, which she still used daily. Urine drug screen was not obtained during admission. The patient had started taking bupropion 150 mg daily 1 month ago with good control of mood symptoms but had discontinued all other psychotropic medications, citing inefficiency. She reported nightly use of her BiPAP machine with good efficacy.

Hospital stay was notable for some tremulousness and anxiety; this was associated with marijuana withdrawal and resolved with initiation of gabapentin 100 mg twice daily. Hydroxyzine 50 mg was available every 6 hr as needed for anxiety. Her 150 mg bupropion daily was continued during admission. Trial of 1 mg risperidone daily for affective lability showed good efficacy within 4 days of initiation. The patient used BiPAP machine and a wedge under her mattress nightly to maximize sleep hygiene. Patient's severity of snoring had decreased since last admission. She had no difficulties falling or staying asleep. She was monitored regularly by the psychiatric and general pediatrics team throughout admission. The patient denied significant depressive symptoms or suicidality at all points during admission. She was enthusiastic about finding an effective medication regimen and continued gabapentin, bupropion, and risperidone at discharge. She was discharged after 5-day stay in good condition. She was interested in scheduling a tonsillectomy in the future, so an otolaryngology consult was placed at time of discharge.

## 3. Discussion

Our patient had long course of poorly controlled psychiatric conditions, multiple inpatient psychiatric stays, and polysubstance abuse in the setting of undiagnosed OSA. Her psychiatric and sleep symptoms both began at age 12, but OSA was not diagnosed until age 15 after multiple inpatient psychiatric hospitalizations. During this diagnostic interim, many psychotropic medications were trialed with little efficacy. After starting treatment for her OSA, both her psychiatric symptoms and her polysubstance use disorder improved markedly.  Table 1 outlines the differences in presentation to our facility before and after management of OSA. This improvement highlights the importance of continuous screening to unveil underlying medical conditions that may be contributing to psychiatric conditions.

Sleep aberrations may be one explanation for the similar symptomatology in OSA and psychiatric conditions such as depression. Generally speaking, poor sleep is associated with increased negative affect, e.g. increased stress, anxiety, and anger [17], and both OSA and depression are associated with poor sleep. One proposed mechanism for this relationship involves suppression of the locus coeruleus, a brainstem nucleus with roles in the brain's noradrenergic system. This nucleus is suppressed primarily during REM sleep, leading to dose-dependent norepinephrine reductions that maintain REM sleep [18]. Decreased norepinephrine leads to less binding to a two receptors of the medial prefrontal cortex (mPFC), decreasing mPFC activity, which in turn disinhibits the amygdala leading to an overall increase stress-pathway signaling. This mechanism is particularly significant in our patient because pediatric populations spend more time in REM sleep [9]. Depression further amplifies this effect because it is independently associated with decreased REM latency (time to enter REM phase), increased REM intensity (number of REM episodes), and increased REM duration [19]. This amplified limbic response has been corroborated by functional magnetic resonance imaging studies [20]. Other studies have also demonstrated similar nonspecific amygdala reactivity in depressed patients [21].

Symptom overlap between OSA and depression may also be explained by connectivity changes between the central executive network (CEN), the salience network (SN), and the default mode network (DMN). The CEN selects relevant stimuli and supports goal-oriented (externally directed) cognition, the SN mediates switching between networks based on relevant stimuli, and the DMN guides responses to stimuli by supporting self-related (internally directed) cognition [22]. The anterior DMN aids the limbic system in self-referential processing and emotion regulation, while the posterior DMN aids the hippocampus in consciousness and memory processing [22]. Literature supports four network-based changes in patient with MDD: increased connectivity within the anterior DMN, increased connectivity between SN and anterior DMN, changed connectivity between anterior and posterior DMN, and decreased connectivity between posterior DMN and CEN [23]. OSA patients share the latter two network changes [24–27]. Thus, the decreased connectivity between posterior DMN and CEN found in both OSA and depression may explain the shared concentration and attention difficulties. The effect of changed connectivity between anterior and posterior DMN is less clear, though it likely contributes to some overall cognitive deficit.

After diagnosis of OSA and management of symptoms with BiPAP machine, the patient reported significant improvement in mental health. To date, one case report details the full resolution of depression in a pediatric patient after palatal expansion surgery resolved comorbid apnea [28]. In our patient, while mood symptoms were still present, they had dramatically decreased in severity and were now responsive to medical management with bupropion and risperidone. This partial improvement in mood is more typically expected when initiating treatment for OSA. A 2013 meta-analysis likewise details both how depressive symptoms are not only more common in pediatric OSA patients but also how appropriate OSA management leads to decreases in symptom burden [29]. Additionally, obese pediatric OSA patients tended to have more significant symptoms of depression compared to nonobese children with OSA [23]. Depression and OSA have overlapping symptoms including decreased energy, fatigue, and difficulty concentrating. Thus, while unimodal treatments in comorbid patients might somewhat decrease the severity of symptoms, the improvement is partial at best and likely pales in comparison to a multimodal approach [30].

Once the patient's OSA was managed, she also discontinued all illicit drug use except marijuana. Very little literature exists discussing the interplay between SUD and OSA, but sleep disturbances and SUDs are known to be linked [19–21]. An analysis of survey data from 13,831 adolescents showed a relationship between substance use and sleep disturbances that appeared strongly associated with psychiatric problems [31]. Another study showed that both fewer hours of sleep and decreased quality of sleep in pediatric males were associated with earlier and repeated use of both alcohol and cannabis [32]. Most recently, a 2023 study reports an independent association between severity of pediatric OSA and substance use [33]. Some postulate substance abuse may serve as a form of self-medication in patient with low mood or sleep problems. Examples of this include the use of alcohol and tranquilizers to promote sleep and use of stimulants to gain energy [2]. While their intentions are understandable, the substance abuse may ultimately aggravate their comorbid conditions, worsening sleep quality, or even inducing apnea, e.g., when using opioids for sleep [34].

The patient's response to previous medications highlights how sleep disturbances secondary to OSA may have played a role in her mood. The only medications that provided symptom relief were bupropion and methylphenidate, both stimulant medications that may have improved the patient's fatigue caused by her underlying OSA. Bupropion is FDA-approved for reducing nicotine cravings, so this medication may have helped manage nicotine cravings [35]. Regarding the methylphenidate, while there may be some apprehension in using stimulant medication in the context of substance abuse, literature shows proper ADHD treatment has long-term protective effects on substance abuse; on the other hand, untreated ADHD may in reality be more concerning as it is associated with increased likelihood of substance use behavior [36, 37].

Prazosin was trialed for sleep without benefit in our patient. This medication is an alpha-1 adrenergic antagonist that can treat PTSD-associated nightmares by decreasing brain activity associated with fear and startle responses [38]. Furthermore, this medication is known to exacerbate OSA symptoms due to reversible inhibition of neurons in the hypoglossal motor nucleus, thereby decreasing tone in genioglossus muscles [39, 40]. This may decrease airway patency and worsen airway obstruction in OSA patients [20].

OSA, depression, and polysubstance abuse share many features, and it may often be challenging to discern the true symptom timeline. Regardless, it is important to maintain a high degree of suspicion for OSA in patients with psychiatric comorbidities and polysubstance abuse, as proper treatment of underlying OSA can considerably improve outcomes for patients with SUD and psychiatric conditions.

## Figures and Tables

**Table 1:** Patient presentation before and after initiation of BiPAP for OSA.

	Before BiPAP use	After BiPAP use
Overnight oxygen saturation	63.5%–88%	>90%

Presenting symptoms	Depressed mood with active suicidal ideation, anxiety, fatigue, diffuse body aches, significant sleep disturbances	Mildly depressed mood, no active suicidal ideations, acute marijuana withdrawal, no sleep complaints

Active substance use	Marijuana, nicotine vapor, intranasal cocaine, psychedelics, daily alcohol use	Marijuana, occasional alcohol use

Medication regimen at admission	Aripiprazole 10 mg, fluoxetine 40 mg, bupropion 150 mg (all discontinued by patient prior to admission)	Active : bupropion 150 mg

Medication regimen at discharge	Lithium 450 mg	Bupropion 150 mg, risperidone 1 mg, gabapentin 100 mg twice daily

## Data Availability

All data underlying the results are available as part of the article and no additional source data are required.
